# An Intercomparison Between NPL (India) and NIST (USA) Pressure Standards in the Hydraulic Pressure Region up to 26 MPa

**DOI:** 10.6028/jres.099.064

**Published:** 1994

**Authors:** J. K. N. Sharma, Kamlesh K. Jain, C. D. Ehrlich, J. C. Houck, D. B. Ward

**Affiliations:** National Physical laboratory, New Delhi, India; National Institute of Standards and Technology, Gaithersburg, MD 20899-0001

**Keywords:** hydraulic pressure, piston gauge, pressure

## Abstract

Results are presented of an intercomparison of pressure measurements between the National Physical Laboratory (NPL), India, and the National Institute of Standards and Technology (NIST), USA, using piston gauge pressure standards over the range 6 MPa to 26 MPa. The intercomparison, using the NPL piston gauge pressure standard, with a nominal effective area of 8.4×10^−5^ m^2^ and the *NIST* piston gauge pressure standard, with a nominal effective area of 2.0× 10^−5^ m^2^ was carried out at the NPL.

The intercomparison data obtained show a relative difference of 1 × 10^−6^ in the zero-pressure effective area (*A*_0_) of the NPL standard as obtained by the NIST standard. At 6 MPa the relative difference in effective areas is 3.5×10^−6^; at the full scale pressure of 26 MPa, the relative difference is 12×10^−6^. These differences are in excellent agreement with the statements of uncertainty of the respective standards as obtained from the primary standards of these two laboratories.

## 1. Introduction

Piston gauges [1] and liquid column manometers [2] are widely used instruments for the most accurate measurement of pneumatic pressure in the near-atmospheric pressure region (10 kPa to 1 MPa). Piston gauges are also used to measure pneumatic pressures to over 100 MPa, and hydraulic pressures from as low as 1 MPa to over 1 GPa. In a typical piston gauge, a cylindrical piston rotates in a closely-fitted cylinder. The pressure is derived from the known downward gravitational force on the piston and weights that is balanced by an upward force generated by the action of the system pressure on a known area when the piston is floating at its reference position. The uncertainty with which a pressure measurement can be made using a piston gauge then depends on the uncertainties with which measurements of both the downward force and the effective area of the piston-cylinder assembly can be made.

The elastic deformation of the piston-cylinder assembly is usually negligible in the atmospheric pressure range. The uncertainty in the evaluation of the effective area at low pressure [3] is mainly limited by the uncertainty with which absolute dimensional measurements can be made of the piston and cylinder. Recent studies [4] have shown that the effective areas of large-diameter (35 mm), atmospheric-pressure range piston-cylinder assemblies may have significant distortion coefficients, at the 6 parts in 10^6^ level. Even so, effective areas of these gauges obtained by dimensional measurements and incorporating theoretical distortion coefficients based on simple elastic theory are found to agree to within 10 parts in 10^6^ with values obtained by crossfloat against either manometers or standard gauges having known pressure dependence of the effective area.

At higher pressures, besides the dimensional uncertainty, there is additional uncertainty in determining the effective area of a piston gauge due to the distortion of the piston and cylinder assembly. Compounding the problem, higher-pressure pistons and cylinders typically have relatively small diameters, so that a given dimensional uncertainty results in a relatively larger uncertainty in both the low-pressure effective area (*A*_0_) and the distortion coefficient (b) of the gauge [5].

Close agreement of experimentally-determined pressure-dependent effective areas, using different techniques within a metrological laboratory, creates confidence in the measurements. To add further confidence in pressure measurement, international intercomparisons are often performed. Such intercomparisons can establish uniformity of measurements and mutual compatibility of standards, and reveal possible systematic errors or reaffirm the uncertainty within which the laboratory can make relative pressure measurements.

While the results of international intercomparisons of piston gauges in the pneumatic pressure region [6, 7] up to 10 MPa, and at hydraulic pressures [8–12] up to 500 MPa, are reported in the literature, there is relatively little such intercomparison in the lower hydraulic pressure region. With this in mind, a series of pressure comparison measurements in the hydraulic hydrostatic pressure region up to 26 MPa was carried out between NPL (India) and NIST (USA). Direct piston gauge crossfloats used to accomplish the comparison were performed at NPL, and the results are reported here.

## 2. Description and Metrological Characteristics of the Standards

### 2.1 The NPL Transfer Standard

**Fig. 1:**
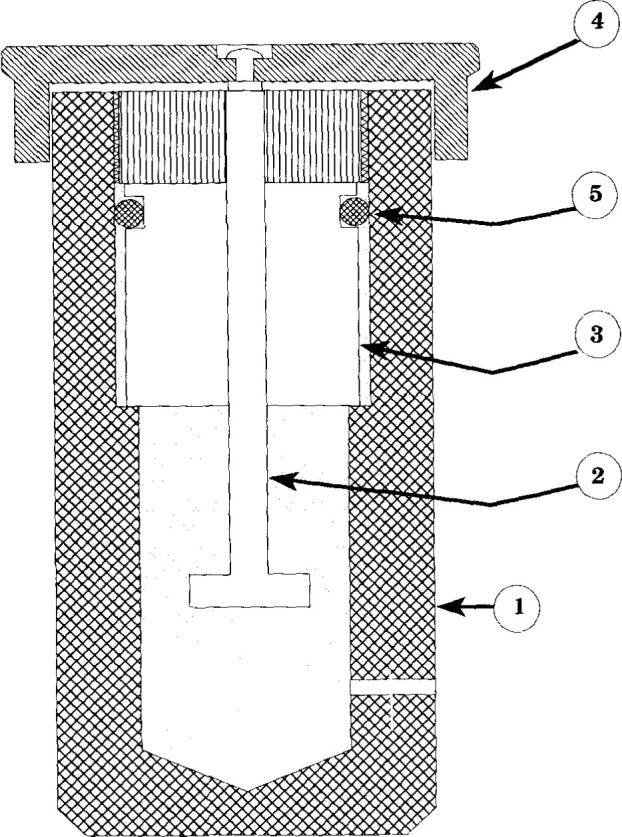
A schematic cross-sectional view of the measuring system of the NPL piston gauge standard NPL-28: (1) pressure column, (2) piston, (3) cylinder assembly, (4) weight table (5) O-rings.

**Fig. 2:**
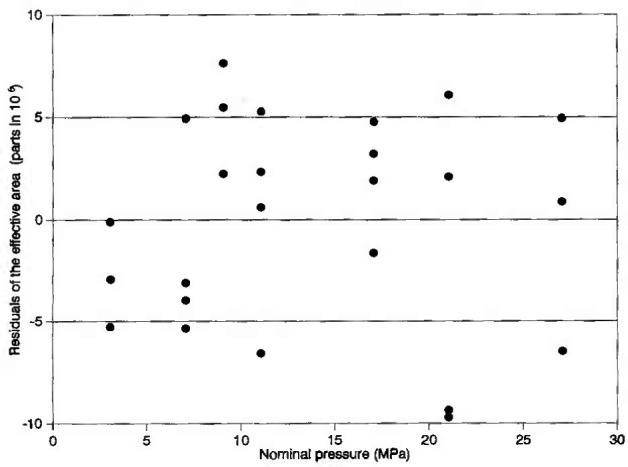
Residuals of measured values of the effective area (*A*_e_) of PL-28 from the best linear fit of the model *A*_e_*=A*_0_(1+*bp*), where *A*_0_=8.400 423×10^−5^ m^2^ and *b*=−1.62×10^−12^ Pa^−1^, obtained when calibrated by the NPL standard (NPL-100).

The NPL transfer standard piston gauge that was used for these measurements, denoted NPL-28, is equipped with a reentrant type piston-cylinder system capable of measuring a full-scale pressure of 28 MPa. A schematic representation of the NPL piston gauge is shown in Fig. 1. The piston is rotated to relieve friction by a pulley coupled to a dc motor. To minimize thermal problems the motor is mounted at a distance of 300 mm from the piston-cylinder assembly. The effective area of NPL-28 was determined by dimensional measurements and also by crossfloating over the range 5 MPa to 26 MPa against another NPL piston gauge of 100 MPa full scale pressure, denoted NPL-100. NPL-100 in turn was calibrated against the NPL controlled clearance primary pressure standard. The other parameters associated with NPL-28 are given in Table 1. Figure 2 shows the residuals from the best linear fit of the effective area *A*_e_ of NPL-28 as a function of the nominal applied pressure, as obtained using NPL-100 as the standard. The best linear fit of the model *A*_e_*=A*_0_(1*+bp*) is obtained when *A*_0_=8.400 423×10^−5^ m^2^ and *b*=−1.62×l0^−12^ Pa^−1^.

The 3*σ* standard deviation of the *A*_0_ coefficient is (6×10^−6^) *A*_0_. The 3*σ* overall uncertainty of *A*_e_ of NPL-28 as obtained during calibration by NPL-100 is (88×10^−6^) *A*_c_.

### 2.2 The NIST Transfer Standard

**Fig. 3:**
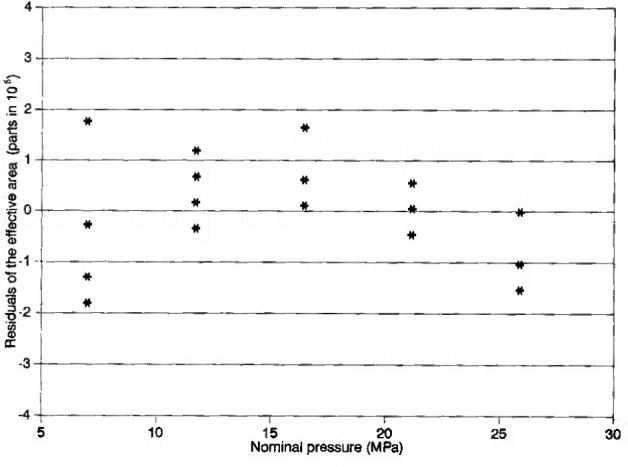
Residuals of measured values of the effective area (*A*_e_) of the NIST piston gauge standard NIST-45 from the best linear fit of the model *A*_e_*=A*_0_(1+*bp*), where *A*_0_=1.961 191×10^−5^ m^2^ and *b*=9.85×10^−13^ Pa^−1^, obtained when calibrated by the NIST primary standard (NIST-27) used in the controlled clearance gauge mode.

The NIST transfer standard piston gauge, denoted NIST-45, is equipped with a simple-type piston-cylinder assembly having a full pressure range of 50 MPa. The piston is rotated by an oval-shaped pulley coupled to a dc motor mounted at a distance from the piston-cylinder in order to minimize the heat transferred from the motor to the piston and cylinder during operation. The effective area and the pressure coefficient of the piston-cylinder assembly of NIST-45 were obtained at NIST [13] by calibrating NIST-45 against primary controlled clearance piston gauge NIST-27, which has a full pressure range of 28 MPa. Figure 3 shows the residuals of the effective area of NIST-45 from the best fit of the model *A*_e_=*A*_0_(1+*bp*) where *A*_0_=1.961 191×10^−5^ m^2^ and *b*=9.85×10^−13^ Pa^−1^. The 3*σ*-uncertainty of *A*_e_ of NIST-45 as obtained during calibration by NIST-27 is (35×10^−6^) *A*_e_.

## 3. Experimental Procedure

The piston gauges used were kept on a heavy non-magnetic stainless steel base to minimize vibration and magnetic effects. All measurements were made in an environment which provided stable temperature conditions of (23±1)°C. The temperature of NPL-28 was measured within 0.1 *°*C by a mercury-in-glass thermometer placed near the pressure column. The temperature of the NIST transfer standard was measured with a platinum resistance thermometer (PRT) attached near the piston, and its output was read with an autoranging digital multimeter having a resolution of 2 mΩ corresponding to a temperature resolution of 0.005 °C.

The intercomparison between NPL-28 and NIST-45 was carried out using the well-established crossfloat method [1]. Before the crossfloat, both piston gauges were leveled to ensure the verticality of their axes, and the systems were checked for leaks to the full scale pressure of 28 MPa. The piston gauges were loaded with the weights calculated to generate the desired pressure, and were then pressurized to float at their reference levels. The gauges were then isolated from the rest of the pressurizing system, and subsequently from each other, by closing the isolation valves provided in the pressure line and between the gauges. The position and fall rate of both pistons were measured using the output of an electronic displacement transducer recorded on a strip chart recorder. By adjusting the fractional weights on NIST-45, which was generating comparatively lower pressures, crossfloat equilibrium was achieved, as determined when both gauges had the same respective fall rates independent of whether the isolation valve between the gauges was closed or open. The pressure was then increased to the next higher step, as discussed later, and the procedure was repeated, up to the pressure of 26 MPa. A period of about 30 min between two successive pressures was found adequate to allow the system to return to equilibrium, and about 10 min was required to ref)eat the observation at any pressure point.

For an individual crossfloat balance, the effective area of the test gauge expressed in terms of the other experimental parameters is [13]:

$$A_{e(T)}=A_{0}\left(1+bp\right)\left[1+\left(\alpha_{p}+\alpha_{c}\right)\left(T-T_{r}\right)\right]$$ (1a)

$$=\frac{\sum_{i=1}^{n}\left[M_{i}\left(1-\frac{\rho_{\text{air}}}{\rho_{m_{i}}}\right)g\right]+\gamma_{f}\cdot C}{p_{s}+\Delta p}$$ (1b)

where

*M_i_*: is the true mass of the *i*th weight on the test gauge
*ρ*_air_: is the density of air in the vicinity of the weights
$\rho_{m_{i}}$: is the density of the *i*th weight on the test gauge
*g*: is the local acceleration due to gravity
*A*_0_: is the effective area at the reference temperature and atmospheric pressure
*b*: is the pressure distortion coefficient of the piston and cylinder combination
*α*_p_: is the linear thermal expansion coefficient of the piston
*α*_c_: is the linear thermal expansion coefficient of the cylinder
*T*: is the temperature of the piston and cylinder
*T*_r_: is the reference temperature
*γ*_f_: is the surface tension of the operating liquid
*C*: is the circumference of the piston
*P*_s_: is the pressure at the reference level of the standard gauge.

Δ*p* is the head correction (*ρ*_f_–*ρ*_air_)*gH*, where *H* is the height difference between the reference levels of the two gauges and *ρ*_f_ is the density of the pressure transmitting fluid. Δ*p* can be positive or negative depending on whether the reference level of the standard is lower or higher than that of the test gauge.

As it was not possible to bring the reference levels of the individual piston gauges to the same operating level during crossfloat, a pressure head correction term (Δ*p*) was applied. In this case, the reference level of NPL-28 was higher by 0.105 m than that of NIST-45.

A computer program developed and used at NIST [7] gives the effective area and the pressure coefficients of the test gauge based upon those of the standard. This program also provides the residuals and the standard deviation of the predicted value of the area, and the standard deviation of the coefficients.

## 4. Results and Discussion

Three test cycles, up to 26 MPa, were carried out during the intercomparison of NPL-28 and NIST-45. In one cycle, the pressure was increased to (6, 12, 16, 20 and 26) MPa, and then decreased from (20 to 6) MPa in similar steps. In the other two cycles, the measurement proceeded from the highest pressure to the lowest and back to the highest. A fourth set of observations was also taken where the pressure was increased from the lowest to the highest pressure only. A total of 32 independent observations were made, nine in each of the first three test cycles and five in the fourth test.

**Fig. 4:**
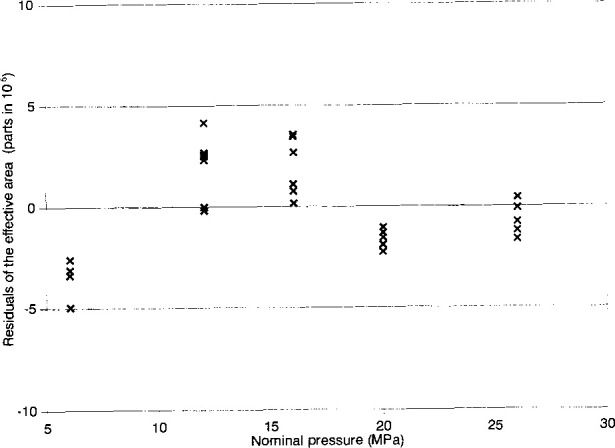
Residuals of measured values of the effective area (*A*_e_) of NPL-28 from the best linear fit of the model *A*_e_*=A*_0_(1+*bp*), where *A*_0_=8.400 415×10^−5^ m^2^ and *b*=−2.05×10^−12^ Pa^−1^, obtained when calibrated by NIST-45.

Figure 4 shows a plot of the residuals of the effective area of NPL-28, as a function of the nominal applied pressure, when NPL-28 is crossfloated against NIST-45. This figure gives the deviation of the measured values of the effective area, in parts in 10^6^, for the individual measured pressures, from the fitted equation *A*_e_=*A*_0_(1+*bp*) where *A*_0_=8.400 415×10^−5^ m^2^ and *b*=−2.05×10^−13^ Pa^−1^. The distribution of the residuals of the effective area (*A*_e_) of NPL-28 in Fig. 4 is taken to be random.

The value of *A*_o_ of NPL-28 as obtained by crossfloat against NPL-100 exceeds by 1×10^−6^ the value obtained by crossfloat against NIST-45. This 1×10^−6^ difference is well below the 3*σ* standard deviation of the *A*_0_ coefficient, and hence the agreement at low pressure is excellent. Further, the value of *b* for NPL-28 when it is crossfloated against NIST-45 differs from the value when it is crossfloated against NPL-100 (given in Table 1) by 0.43×10^−12^ Pa^−1^. Considering the 3*σ* standard deviations of these measured values, i.e., 0.2×10^−12^ Pa^−1^ and 0.4×10^−12^ Pa^−1^, respectively, the difference is not unreasonable. Additionally, these observed differences in *A*_0_ and *b* cause a relative difference in the effective area of 3.5×10^−6^ at a measured pressure of 6 MPa, increasing to 12×10^−6^ at a full scale pressure of 26 MPa. These results are compatible with the measurement uncertainties associated with the individual piston gauges as given in Table 1.

The uncertainty in the measurement of pressure using a piston gauge arises from two main sources: (1) inherent uncertainties associated with the gauge itself and (2) other uncertainties associated with the local experimental conditions. The former is mainly attributable to the determination of the effective area of the piston-cylinder assembly and uncertainties in the mass of the load and/or piston. However, the latter arises from the experimental procedures, the major components of which were (i) uncertainty associated with the measurement of temperature, (ii) correction due to any difference of reference levels and (iii) the resolution of the balancing criteria when the two systems are in equilibrium.

During the cross float of NPL-28 and NIST-45 the fractional mass was adjusted so as not to contribute more than ± 1.2×10^−6^ uncertainty (3*σ*) at the minimum pressure of 6 MPa, which decreases to less than 1×10^−6^ at the full scale pressure of 26 MPa. As the reference levels were measured with an uncertainty of 5.00× 10^−4^ m (3*σ*) and temperature was read with an estimated accuracy of 0.1 °C (3*σ*), the contribution to the total estimated uncertainties in effective area due to temperature and difference in reference level is not significant compared to the total uncertainties associated with the standards NPL-28 and NIST-45

## 5. Conclusions

The comparison of the effective area of NPL-28 as determined by NIST-45 and NPL-100 show agreement between the two pressure standards (NPL-28 and NIST-45) that is significantly better than the estimated (3*σ*) uncertainty of either gauge. The low-pressure area of NPL-28 obtained from NPL-100 differs by only 1×10^−6^ from the area value obtained during comparison with NIST-45. The effective areas of NPL-28 determined by these same two paths differ by 3.5×10^−6^ at 6 MPa, increasing to 12×10^−6^ at 26 MPa. This study thus shows the agreement of measurements of effective area, and hence demonstrates the compatibility of the standards maintained by these two laboratories, for hydraulic pressures to 26 MPa.

## Figures and Tables

**Table 1:** Description and metrological parameters of the piston-cylinder assemblies used in the prcssuie comparison measurements

Piston gauge designation	NPL-28	NIST-45
Piston-cylinder (type)	Reentrant	Simple
Full scale pressure (MPa)	28	50
Piston material	Tungsten carbide	Tungsten carbide
Cylinder material	Tungsten carbide	Tungsten carbide
Fluid	Spinesstic 22^a^	Spinesstic 22^a^
Coefficient of thermal expansion for piston (°C^−1^)	4.5×10^−6^	4.5×10^−6^
Coefficient of thermal expansion for cylinder (°C^−1^)	4.5×10^−6^	4.5×10^−6^
Effective area at atmospheric pressure and at 23 °C (m^2^)	8.400 423×10^−5^	1.961 191×10^−5^
Distortion coefficient (Pa^−1^)	−1.62×10^−12^	9.85×10^−13^
Estimated total relative uncertainty (3*σ*) of the effective area, Δ*A*_e_/*A*_e_	88×10^−6^	35×10^−6^

a Certain commercial equipment, instruments, or materials are identified in this paper to foster understanding. Such identification does not imply recommendation or endorsement by the National Institute of Standards and Technology, nor does it imply that the materials or equipment identified are necessarily the best available for the purpose.
